# Impact of Altitudinal Variation on the Phytochemical Profile, Anthelmintic and Antimicrobial Activity of Two *Pinus* Species

**DOI:** 10.3390/molecules26113170

**Published:** 2021-05-26

**Authors:** Wafaa M. Elkady, Mariam H. Gonaid, Miriam F. Yousif, Mahmoud El-Sayed, Hind A. N. Omar

**Affiliations:** 1 Department of Pharmacognosy, Faculty of Pharmacy, Future University in Egypt, Cairo 12311, Egypt; mariam.hussein@fue.edu.eg (M.H.G.); miriam.fouad@fue.edu.eg (M.F.Y.); 2 Microbiology Department, Faculty of Veterinary Medicine, Suez Canal University, Ismailia 41522, Egypt; Mahmoud_elsayed@vet.suez.edu.eg; 3 Forestry and Range Sciences Department, Faculty of Natural Resources and Environmental Sciences, Omar Al-Mukhtar University, Al Bayda’ 00218-84, Libya; jehadalsaadi1982@gmail.com

**Keywords:** essential oils, environmental conditions, GC/MS, *Pinus halepensis* L., *Pinus pinea* Mill

## Abstract

Active components from natural sources are the current focus in most pharmacological research to provide new therapeutic agents for clinical use. Essential oils from the *Pinus* species have been traditionally used in medicine. This study aimed to investigate the chemical profile of two *Pinus* species, *Pinus halepensis* L. and *Pinus pinea* Mill, from different altitudes in Libya and study the effect of environmental conditions on the biological activities of essential oils. A clevenger apparatus was used to prepare the essential oils by hydrodistillation. Analyses were done using GC/MS. Anthelmintic and antimicrobial activities were tested against the earthworm *Allolobophora caliginosa*, gram-positive bacteria, gram-negative bacteria, and fungi. Different chemical profiles were observed among all tested essential oils, and terpenes were the most dominant class. All studied essential oils from the *Pinus* species exhibited a remarkable anthelmintic activity compared to the standard piperazine citrate drug. *Pinus halepensis* from both altitudes showed broad-spectrum antimicrobial activity against all tested microorganisms, while *Pinus pinea* was effective against only *Escherichia coli*. From these findings, one can conclude that there are variations between studied species. The essential oil compositions are affected by environmental factors, which consequently affect the anthelmintic and antimicrobial activity.

## 1. Introduction

Throughout history, medicinal plants have been used for the curing of many diseases. They have been considered valuable sources for the development of novel therapies [1]. Essential oil has been widely studied for different biological activities and uses in pharmaceutical industries. Essential oils from many *Pinus* species have been widely used in the drug, food, and cosmetic industries [2,3].

Genus *Pinus* (Pinaceae) includes 70–100 species. They are evergreen trees grown as ornaments and timber trees as well as for their oleoresin, which upon distillation, yields turpentine or pine tar (colophony resin) and turpentine oil [4,5]. Pine products have acquired great commercial importance and have been long used in traditional medicine. Pine tars and turpentine oils are usually used externally as a rubefacient or counterirritant for rheumatic ailments, as an analgesic, and to treat skin diseases such as eczema, while pine needle oils have been used as inhalants for nasal decongestant, as expectorant, and to relieve cough [2,5].

*Pinus halepensis* and *Pinus pinea* are among the *Pinus* species that are cultivated in Libya; both are native to Mediterranean regions [5]. All parts of the *P. halepensis* (Aleppo pine) tree possess potential medicinal values [6]. The tree has several phytoconstituents as phenolic compounds, terpenes, and turpentine that have many valuable therapeutic applications [7,8]. *P. pinea* (stone pine or umbrella pine) were usually cultivated for their edible pine nuts. Their essential oil is usually used for many skin ailments and for herbal steam baths (inhalers) for respiratory problems. Resins were used as antiseptics and for kidney remedies [9,10].

Reports have shown that the main volatile constituents usually found in P. halepensis are β-caryophyllene, α-humulene, and aromadendrene. However, *P. pinea* is characterized by the presence of limonene β-phellandrene and α-pinene [11].

Several studies have previously dealt with the constituents of *P. halepensis* and *P. pinea* needles, but the composition of the essential oil usually varies due to the geographic location, climatic conditions, and the time of collection; this may also affect its biological potential [4,9,12,13]. To the best of our knowledge, no comparative study has been published related to the chemical composition and anthelmintic and antimicrobial activities of *P. halepensis* and *P. pinea* essential oils in Libya, reflecting the impact of geographic difference and ecological conditions. Therefore, this work aims to study the impact of different altitudes on the phytochemical composition of the two species from different localities in Al-Jabal Al-Akhdar (Libya) and to study the effect of these phytochemical variations on the anthelmintic activity against the earthworm *Allolobophora caliginosa* and the antimicrobial activity against the yeast pathogen *Candida albicans,* as well as against gram-positive and gram-negative bacteria.

## 2. Results

### 2.1. GC/MS Analysis of the Essential Oil

Hydrodistillation of the aerial parts of *P. halepensis* and *P. pinea* from different localities vary in the yielded content. *P. halepensis* aerial parts showed a high percentage yield of Ph-1 (0.22%) (*P. halepensis* at altitude 830 m) and Ph-2 (0.59%) (*P. halepensis* at altitude 75 m), which was more than *P. pinea*, which yielded Pp-1 (0.12%) (*P. pinea* at altitude 625 m) and Pp-2 (0.22%) (*P. pinea* at altitude 408 m). The low altitude in both studied species (Ph-2 and Pp-2) showed a relatively higher percentage yield (Table 1). These results were in agreement with a previous study in Algeria [4]. This could demonstrate that low altitudes have more preferable environmental conditions for essential oil production in *Pinus*. All isolated essential oils were faint yellow in color and their odors were characteristic.

**Figure 1:**
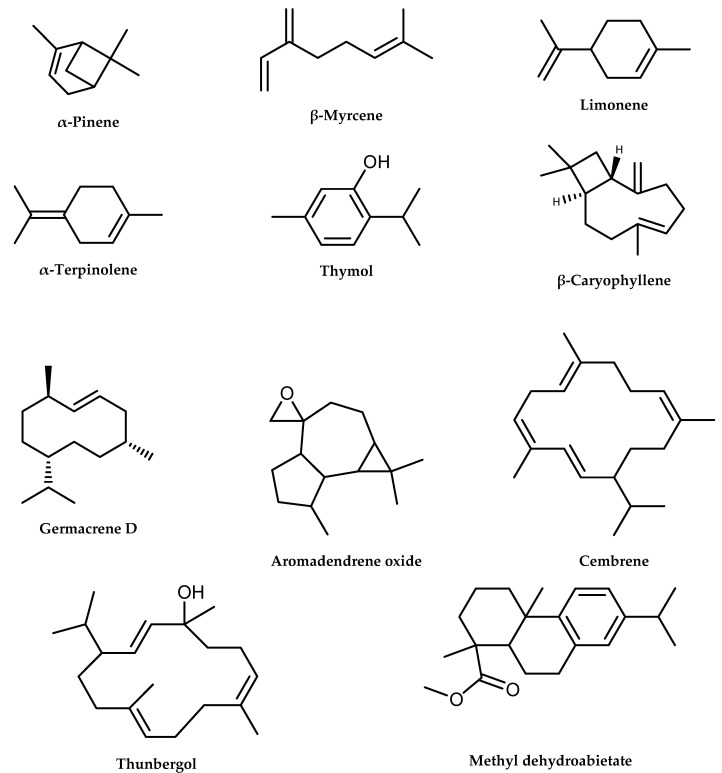
Structures of the major/marker constituents of *P. halepensis* (Ph-1 and Ph-2) and *P. pinea* (Pp-1 and Pp-2) essential oils.

Results are presented in Table 1, the most prominent components of which are shown in bold. The chemical structures of the major constituents of each essential oil are displayed in Figure 1.

### 2.2. In Vitro Anthelmintic Activity of the Essential Oils

**Figure 2:**
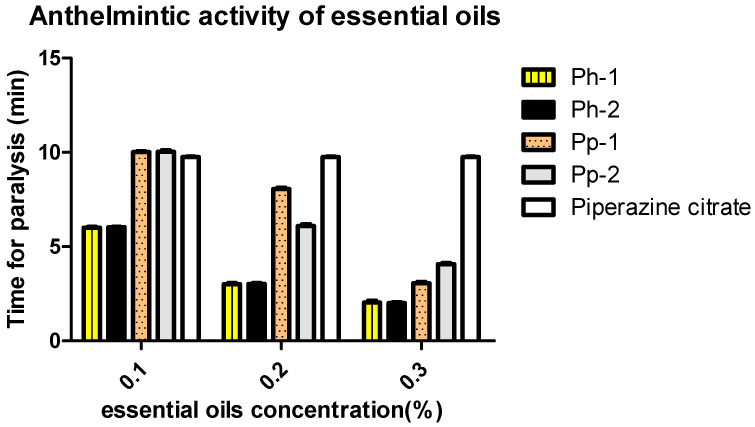
Anthelmintic activity of *P. halepensis* (Ph-1 and Ph-2) and *P. pinea* (Pp-1 and Pp-2) essential oils. Values are expressed as the means ± SD of the inhibition zone of three independent experiments.

The prepared essential oils from both *Pinus* species displayed a dose-dependent inhibition of motility (paralysis) for adult earthworms; the time required to produce such paralysis was calculated in minutes. The essential oils from *P. halepensis* Ph-1 and Ph-2 showed the most potent inhibitory effect compared to that obtained from *P. pinea* Pp-1 and Pp-2, as shown in Figure 2. Remarkably, the influence of higher concentrations (0.3%) of Ph-1 and Ph-2 on worm paralysis was greater than that produced by the positive control, which was treated with piperazine citrate. Statistical analysis revealed no significant differences between the results obtained from the same species. On the other hand, there was a significant difference between the results obtained from the different species under investigation. Results showed that all essential oils from both *Pinus* species could eradicate worms in a short time compared to the reference drug piperazine citrate.

### 2.3. In Vitro Antimicrobial Activity of the Essential Oils

Results revealed that the essential oils obtained from *P. halepensis* possessed stronger and more significant antimicrobial effects against the tested gram-positive, gram-negative, and fungi when compared to that of *P. pinea*.

Essential oils of *P. halepensis* from both localities (Ph-1 and Ph-2) displayed effective antimicrobial activity against the tested gram-positive bacteria (*Bacillus subtilis* and *Micrococcus lutea*). However, moderate activity was observed against the tested gram-negative bacteria (*Proteus mirabilis* and *E. coli*). It also showed moderate antifungal activity against *C. albican* fungi. The essential oils from the low altitude locality (Ph-2) demonstrated greater activity than Ph-1 (Table 2).

A sensitivity test showed that cefotaxime, amikacin, and ceftriaxone had a significant effect against the different tested microorganism strains. As cefotaxime was found to have the greatest inhibition, it was considered as a reference drug for the evaluation of the antibacterial activity. The reference drug for the antifungal activity was nystatin (Table 3).

On the other hand, *P. pinea* essential oils (Pp-1 and Pp-2) showed moderate activity against *E. coli* only, with no obvious effect against either the tested gram-positive bacteria or fungi. The variation in chemical constituents of the essential oil could be responsible for the different antimicrobial effects.

## 3. Discussion

*Pinus* is one of the conifers genera. It is considered one of the main sources of essential oils all over the world [4]. Essential oils have several medicinal properties that could be useful in the pharmaceutical field. Environmental factors have a great impact on the essential oil yield, composition, and, consequently, its biological activity [4,13]. This can be recognized from different earlier reports [6,14,15,16,17,18,19].

In the present study, the aerial parts of *P. halepensis* and *P. pinea* from different altitudes were collected. The prepared essential oils were analyzed. Comparing the results in this study with previous reports from different countries, we discovered that there were qualitative and quantitative differences in phytochemical composition. These variations have an impact on the tested biological activity. The results demonstrate that low altitude has an impact on the yield of the essential oil (Ph-2 and Pp-2).

GC and GC/MS analyses led to the identification of 48 constituents (Table 1). Monoterpene and sesquiterpene hydrocarbons are noted as the major classes of compounds in all four studied essential oils, especially in *P. pinea* (Pp-2) (36.27 and 33.16%, respectively). On the other hand, *P. pinea* from the high altitude (Pp-1) is found to be rich in oxygenated monoterpenes (23.36%). Essential oils from both localities for *P. halepensis* (Ph-1 and Ph-2) are rich in oxygenated diterpenes (33.99 and 22.78%, respectively).

The essential oil from *P. halepensis* resulted in the identification of 24 constituents in Ph-1, accounting for 89.39% of the total volatile oil composition. The main identified volatile compounds were α-pinene (8.54%), methyl dehydroabietate (7.78%), and β-myrcene (5.80%). In Ph-2, 14 compounds were identified, accounting for 82.04% of the total volatile oil composition. Thunbergol (18.85%), α-pinene (13.33%), and aromadendrene oxide (10.84%) were the most prominent constituents. On the other hand, 42 compounds were identified in *P. pinea* (Pp-1), accounting for 94.03% of the total volatile oil composition, characterized by the presence of α-pinene (13.82%), thunbergol (6.16%), and α-terpinolene (5.67%). Finally, 28 constituents were identified in Pp-2, accounting for 91.10% of the total volatile oil composition and showing α-pinene (20.97%), limonene (8.49%), germacrene D (7.81%) as the main constituents.

Anthelmintic remedies of plant origin will aid in the improvement of phytotherapeutic products, which are cost effective, nontoxic, and more accessible. In this study, all tested essential oils showed considerable anthelmintic activity against the earthworm *Allolobophora caliginosa.* The greatest activity was observed in the essential oil prepared from *P. halepensis* at a high altitude (Ph-1). This could be due to the presence of lipophilic compounds (monoterpene and sesquiterpene hydrocarbons), which have a great affinity to cell membranes, their inclusions prompting changes in the physicochemical properties of the membrane. Furthermore, oxygen-containing terpenes were found to be more effective than hydrocarbon terpenes [20]. Thunbergol (18.85%) and aromadendrene oxide (12.13%) are the major oxygen-containing terpenes in *P. halepensis*. The anthelmintic activity could be due to their presence as well as the synergistic activity of all other constituents.

A serious health problem is bacterial resistance to multiple antibiotics. Many reports have stated that essential oils are potential sources to produce novel antimicrobial compounds. In several reports, testing the individual essential oil component does not reproduce the same antimicrobial result as the whole one. For that reason, it is doubtful to attribute the biological activity of essential oil to a particular constituent. Bioactivity could be due to the synergistic effects of all components [21].

The antimicrobial activity was more recognizable in *P. halepensis* than in *P. pinea*. Results showed that gram-positive bacteria (*Bacillus subtilis* and *Micrococcus lutea*) are more sensitive to the tested essential oils than gram-negative bacteria (*Proteus mirabilis* and *E. coli*). Most essential oils showed the same effect [19]. This could be related to the nature of the gram-positive bacteria’s outer membrane, which is composed of hydrophobic substances. On the other hand, the gram-negative bacteria’s outer membrane is composed of hydrophilic constituents [19]. Terpenes and oxygenated terpenes which are more prominent in the *P. halepensis* essential oil (Table 1) can penetrate the hydrophobic outer membrane of gram-positive bacteria [19,22]. The antimicrobial activity is usually due to the synergetic effect of all constituents; however, α-pinene is one of the major terpenes that present in all tested essential oils, and thus could have a great role in this activity [19].

## 4. Materials and Methods

### 4.1. Plant Material

Aerial parts (fresh needles) of *P. halepensis* and *P. pinea* were collected during April 2018 from different localities with different altitudes above sea level. The geographical coordinates of sampling sites are presented in Table 4.

The plants’ identity and authentication were done by Mr. Mossa Al-Seayti (Plant Taxonomy Department, Faculty of Science, Omar Al Mokhtar University, Al Bayda, Libya). Voucher samples (Ph-1, Ph-2, Pp-1, and Pp-2) were kept at the Faculty of Pharmacy, Omar Al-Mukhtar University. All plant samples were shade dried, powdered, and stored at a low temperature in closed containers until use.

### 4.2. Essential Oils Isolation

Aerial parts of *P. halepensis* and *P. pinea* from each altitude were separately hydro-distillated using a Clevenger apparatus for 6 h (100 g plant in 0.5 L distilled water). These preparations were carried out in triplicate for each plant. The percentage yield was estimated as the volume (ml) of essential oil for each 100 g of the studied plant. Oils were dehydrated over anhydrous Na_2_SO_4_ and retained in dark glass sealed vials until further analyses.

### 4.3. GC/MS Analyses

GC analysis was done for each essential oil using an Agilent 6890 (Agilent Technologies, Palo Alto, CA, USA). It was equipped with an HP-5MS column (30 m, 0.320 ID, 0.25µm film thickness). Helium carrier gas was used with a flow rate of 1 mL/min. The programmed temperature was applied, starting with 40 °C for 3 min and then gradually increased by 8 °C/min until 250 °C. A total of 1 μL of each essential oil was injected individually at a split ratio of 1/15. The temperatures of the injector and detector were kept at 250 °C and 280 °C, respectively. The relative amounts of the essential oil components were expressed as percentages attained by peak area normalization. An agilent mass selective detector was used for GC/MS analysis in all studied essential oils. The MS functioning parameters were: interface temperature: 280 °C; ion source temperature: 200 °C; EI mode: 70 eV; scan range: 35–500 amu.

### 4.4. Identification of Essential Oil Components

Identification depended on the retention indices (RI), the comparison of their mass spectra with NIST-11 and Wiley library databases (accessed on 18 May 2021), and the published data in the literature [23]. A homologous series of *n*-alkanes (C8–C28) were injected under the same conditions to measure the relative retention indices.

### 4.5. In Vitro Anthelmintic Activity of the Essential Oils

Some intestinal roundworms that can infect the human body are physiologically similar to the earthworm *Allolobophora caliginosa*, which was chosen as a model for the anthelmintic activity [24].

The anthelmintic activity study was done using three different doses of each essential oil (1ml of 0.1%, 0.2%, and 0.3% *v*/*v* in 1% aqueous tween 80) against the earthworm *Allolobophora caliginosa* [1,21]. Briefly, an Anthelmintic test was applied on 24 worms. They were separated into 3 sets; each set had 6 worms (the length of each worm was greater than or equal to 10 cm) and each set was treated with a certain dose of the prepared essential oils. The control group was prepared by 1% aqueous tween 80. The standard anthelmintic drug, piperazine citrate (Sigma-Aldrich, St. Louis, MO, USA), was prepared as a 0.1% solution in tween 80. Piperazine citrate was used as a reference drug. The time required for complete inhibition of the worm response to external stimuli (death) was recorded. This was done by monitoring the absence of any type of worm movement after treatment with prepared essential oils. All essential oils and the reference drug solutions were freshly prepared.

### 4.6. In Vitro Antimicrobial Activity of the Essential Oils

Different prepared essential oils were tested for their antimicrobial activity. The paper disc diffusion method was applied [25]. Briefly, bacterial inoculums were prepared and spread on nutrients agar plates. Sterile filter papers (6 mm diameter) containing 5 µl of each essential oil were placed using sterile forceps on the surface of the inoculated agar plate along with the positive control: ciprofloxacin (5 µg) for the antibacterial activity, nystatin (100 units) for the antifungal activity, and the negative control (10% DMSO in distilled water). All plates were incubated at 37 °C for 24 h. The zone of inhibition was calculated. The experiment was repeated three times for each bacterium culture and compared to the cefotaxime reference standard for the antibacterial activity and the nystatin for the antifungal activity (Wyeth, NJ, USA).

Standard reference strains were used to assess the antimicrobial activity (American Type Culture Collection “ATCC” for bacteria and fungi). The gram-positive bacteria used were *Bacillus subtilis* (ATCC 6051) and *Micrococcus lutea* (ATCC 4698), while the examined gram-negative bacteria were *Escherichia coli* (ATCC 8739) and *Proteus mirabilis* (ATCC 7002). The tested fungal microorganism was *Candida albicans* (ATCC 10231). The microbial inoculate and bacterial and fungal cultures were prepared as suspensions in Roux bottles. Trypticase soy agar (TSA) and Sabouraud dextrose agar (SDA) were used as media. This was done according to the guidelines of the manufacturer (Sigma, St. Louis, MO, USA).

A sensitivity test was carried out according to NCCLS (1997) [26] to assess the sensitivity of different strains of microorganisms to different types of antibiotics.

## 5. Conclusions

In conclusion, the current study highlighted the impact of different altitudes on essential oil chemical composition and the biological activity of two *Pinus* species from different localities in Libya. The low altitude (near the sea level) species showed relatively more preferable conditions for the production of more essential oil yield. All tested essential oils showed considerable anthelmintic activity. The antimicrobial activity in *P. halepensis* from the low altitude (Ph-2) was the most recognized. Additional investigation is required to study the effects of other environmental factors and the subsequent effects on the biological activity of essential oils.

## Figures and Tables

**Table 1:** Chemical composition of the essential oils of *P. halepensis* (Ph-1 and Ph-2) and *P. pinea* (Pp-1 and Pp-2).

	Chemical Name	Retention Index (RI)		Percentages (%)			
				*Pinus halepensis*		*Pinus pinea*	
		AI	RI	Ph-1	Ph-2	Pp-1	Pp-2
	***Monoterpene***						
1.	α-Pinene	932	939	**8.54** ± 0.50 ^a^	**13.33** ± 0.21 ^b^	**13.82** ± 0.03 ^b^	**20.97** ± 0.16 ^c^
2.	Sabinene	970	976	3.15 ± 0.20	−	−	−
3.	β-pinene	974	980	4.08 ± 0.31	−	−	−
4.	β-Myrcene	990	990	**5.80** ± 0.05 ^a^	−	−	1.37 ± 0.06 ^b^
5.	δ-3-carene	1008	1011	4.07 ± 0.19	−	−	−
6.	p-Cymene	1022	1022	1.39 ± 1.10 ^a^	-	0.82 ± 0.21 ^a^	0.69 ± 0.11 ^a^
7.	Limonene	1024	1029	1.49 ± 0.02 ^a^	**6.77** ± 0.02 ^b^	1.13 ± 0.14 ^a^	**8.49** ± 0.01 ^b^
8.	γ-terpinene	1054	1055	3.38 ± 0.19 ^a^	−	3.18 ± 0.09 ^a^	−
9.	α-Terpinolene	1018	1088	1.08 ± 0.05 ^a^	−	**5.67** ± 0.12 ^b^	4.75 ± 0.21 ^b^
				27.18	20.1	24.62	36.27
	***Oxygenated monoterpenes***						
10.	α-Campholenal	1130	1128	−	−	1.85 ± 0.02 ^a^	0.63 ± 0.02 ^a^
11.	*trans*-pinocarveol	1135	1136	−	−	4.89 ± 0.25 ^a^	1.08 ± 0.12 ^b^
12.	Pinocarvone	1160	1164	−	−	1.77 ± 0.31 ^a^	1.35 ± 0.05 ^a^
13.	Borneol	1165	1168	−	−	2.47 ± 0.42 ^a^	0.91 ± 0.14 ^a^
14.	Myrtenol	1194	1195	−	−	1.20 ± 0.29	-
15.	Verbenone	1204	1206	−	−	0.56 ± 0.00	-
16.	*trans*-(+)-carveol	1215	1219	−	−	1.04 ± 0.17	-
17.	Carveol methyl ether	1229	1229	−	−	1.09 ± 0.07	-
18.	Carvone	1239	1236	−	−	0.57 ± 0.12	-
19.	Bornyl acetate	1287	1285	−	−	1.49 ± 0.06 ^a^	1.29 ± 0.07 ^a^
20.	Thymol	1232	1232	−	−	**5.53** ± 0.05 ^a^	0.65 ± 0.01 ^b^
21.	Carvacrol	1298	1298	−	−	0.90 ± 0.23 ^a^	2.08 ± 0.00 ^a^
				Zero	Zero	23.36	7.99
	***Sesquiterpene hydrocarbons***						
22.	α-Cubebene	1345	1348	−	−	1.42 ± 0.09 ^a^	2.64 ± 0.02 ^a^
23.	α-longipinene	1350	1350	3.47 ± 0.19 ^a^	−	2.80 ± 0.01 ^a^	2.64 ± 0.19 ^a^
24.	β-Elemene	1389	1390	−	2.06 ± 0.07 ^a^	1.26 ± 0.05 ^a^	−
25.	β-Caryophyllene	1417	1417	5.29 ± 0.00 ^a^	6.20 ± 0.19 ^a^	1.65 ± 0.12 ^b^	**5.39** ± 0.01 ^a^
26.	Aromadenderene	1439	1430	1.61 ± 0.12 ^a^	1.38 ± 0.12 ^a^	1.01 ± 0.42 ^a^	−
27.	α-Humulene	1452	1454	3.12 ± 0.05 ^a^	2.0 ± 0.29 ^a^	1.19 ± 0.41 ^b^	3.39 ± 0.20 ^a^
28.	Germacrene D	1484	1485	−	0.68 ± 0.02 ^a^	−	**7.81** ± 0.03 ^b^
29.	δ-amorphene	1511	1507	−	−	1.82 ± 0.57 ^a^	4.54 ± 0.11 ^b^
30.	Cis-α-bisabolene	1508	1508	−	−	1.38 ± 0.67 ^a^	0.65 ± 0.09 ^a^
31.	γ-cadinene	1514	1514	−	−	0.21 ± 0.03	−
32.	δ-cadinene	1523	1522	2.63 ± 0.01 ^a^	2.03 ± 0.09 ^a^	4.54 ± 0.07 ^b^	2.62 ± 0.02 ^a^
				16.12	14.35	19.95	33.16
	***Oxygenated sesquiterpenes***						
33.	Caryophyllene oxide	1582	1581	1.39 ± 0.03 ^a^	4.45 ± 0.03 ^b^	1.70 ± 0.54 ^a^	3.55 ± 0.07 ^b^
34.	Aromadendrene oxide	1595	1595	2.37 ± 0.10 ^a^	**12.13** ± 0.36 ^b^	3.63 ± 0.21 ^a^	1.57 ± 0.08 ^a^
35.	Cubenol	1618	1616	−	−	0.89 ± 0.15 ^a^	0.92 ± 0.15 ^a^
36.	Muurolol	1640	1642	−	−	2.59 ± 0.00 ^a^	2.95 ± 0.09 ^a^
37.	Vulgarol B	1688	1688	−	−	2.55 ± 0.02 ^a^	1.74 ± 0.01 ^a^
				3.76	16.58	11.36	10.73
	***Diterpene hydrocarcons***						
38.	Cembrene	1937	1932	2.54 ± 0.05 ^a^	**8.23** ± 0.07 ^b^	2.10 ± 0.02 ^a^	−
				2.54	8.23	2.1	−
	***Oxygenated diterpenes***						
39.	Manool oxide	1987	1994	3.94 ± 0.02 ^a^	2.55 ± 0.25 ^b^	5.06 ± 0.00 ^a^	2.25 ± 0.15 ^b^
40.	Thunbergol	2047	2047	**5.76** ± 0.58 ^a^	**18.85** ± 0.17 ^b^	**6.16** ± 0.02 ^a^	−
41.	1,4-menthano-azulene	2110	2110	−	−	2.67 ± 0.09 ^a^	3.48 ± 0.07 ^a^
42.	Dehydro abietinal	2279	2279	4.14 ± 0.10	−	−	−
43.	Kaur-16-en-19-ol	2346	2346	3.28 ± 0.21	−	−	−
44.	Methyl dehydroabietate	2359	2354	**7.78** ± 0.03 ^a^	−	0.71 ± 0.19 ^b^	−
45.	Dehydro abietic acid	2380	2380	5.74 ± 0.31 ^a^	1.38 ± 0.27 ^b^	0.71 ± 0.30 ^b^	−
46.	Abietic acid methyl ester	2377	2380	3.35 ± 0.06 ^a^	−	0.84 ± 0.09 ^b^	−
				33.99	22.78	12.64	2.25
	***Miscellaneous compounds***						
47.	2-pentadecanone	1694	1698	−	−	1.59 ± 0.01 ^a^	0.70 ± 0.21 ^a^
48.	Pentadecanal	1716	1716	−	−	1.91 ± 0.09	−
				−	−	4.34	0.7
Total %				89.39 ± 4.10	82.04 ± 2.16	94.03 ± 6.75	91.1 ± 2.46
Yield% (*v*/*w*)				0.22	0.59	0.12	0.22

(Ph-1): *P. halepensis* at altitude 830 m; (Ph-2): *P. halepensis* at altitude 75 m; (Pp-1): *P. pinea* at altitude 625 m; (Pp-2): *P. pinea* at altitude 408 m. AI, Kovats index determined experimentally on HP-5MS column relative to C8–C28 *n*-alkanes. RI, Published Kovats retention indices. The main compounds are in bold. The values shown in this table were the average of three replicates and given as mean ± SD (*n* = 3). One-way ANOVA followed by Duncan’s multiple range test were used. Values with different superscripts (a–c) were significantly different at *p* < 0.05. Values followed by a common letter in columns were not significant (*p* > 0.05).

**Table 2:** Antimicrobial activity of the essential oils of *P. halepensis* (Ph-1 and Ph-2) and *P. pinea* (Pp-1 and Pp-2).

	Inhibition Zones	Diameter of Inhibition in mm					
Micro- Organisms		*P. halepensis*		*P. pinea*		Cefotaxime	Nystatin
		Pp-2	Pp-1	Ph-2	Ph-1		
Gram-Positive							
*Bacillus subtilis*		8 ± 0.32	18 ± 0.91	−	−	10 ± 0.89	
*Micrococcus lutea*		39 ± 0.54	39 ± 0.83	−	−	27 ± 0.93	
Gram-Negative							
*Escherichia coli*		10 ± 0.51	18 ± 0.90	18 ± 0.84	8 ± 0.36	27 ± 0.13	
*Proteus mirabilis*		14 ± 0.56	15 ± 0.71	−	−	25 ± 0.83	
Fungi							
*Candida albican*		10 ± 0.25	15 ± 0.13	−	−		18 ± 0.34

Values are expressed as the means ± SD of the inhibition zone of three independent experiments.

**Table 3:** Sensitivity of the tested microorganisms to different antibiotics.

	Gram Negative		Gram Positive		Fungi
	*Bacillus subtilis*	*Micrococcus* *Lutea*	*E. coli*	*Proteus mirabilis*	*Candida albicans*
Cefotaxime	10	27	27	25	−
Erythromycin	26	14	−	−	−
Polymyxin-B	11	18	12	−	−
Cephalexin	10	21	−	−	−
Amoxicillin	−	−	−	15	−
Tetracycline	27	29	21	−	−
Streptomycin	20	27	−	15	−
Nalidixic Acid	20	17	−	20	−
Fusidic Acid	23	20	−	-	−
Amoxyclav	11	14	−	25	−
Carbenicillin	9	11	−	28	−
Gentamicin	22	28	19	20	−
Chloramphenicol	15	23	23	−	−
Amikacin	27	21	19	21	−
Ceftriaxone	12	20	27	20	−
Nystatin					18

Values are expressed as the means of inhibition zones of three independent experiments.

**Table 4:** The geographical coordinates of sampling sites of *P. halepensis* (Ph-1 and Ph-2) and *P. pinea* (Pp-1 and Pp-2).

	Localities	Altitude (m)	Geographical Coordinates	
*P. halepensis (Ph-1)*	Sidi Alhamry	830 m	32°38′286″ N	21°48′164″ E
*P. halepensis (Ph-2)*	Alaslab	75 m	32°54′458″ N	22°09′587″ E
*P. pinea (Pp-1)*	Werdama	625 m	32°47′334″ N	21°46′313″ E
*P. pinea (Pp-2)*	Al-Mansura	408 m	32°50′10″ N	21°51′10″ E

## Data Availability

The data presented in this study are available within the article.

## Sample Availability

Samples of the essential oil are not available from the authors.
